# Contextual analysis of antibiotic prescribing practices for upper respiratory infections and diarrhoea in rural Punjab, Pakistan

**DOI:** 10.1186/s13756-026-01700-3

**Published:** 2026-02-11

**Authors:** Muhammad Ahmar Khan, Nida Khan, Rabab Sakina, Mashal Amin, Shaheer Ellahi Khan, Farah Zafar, Saad Alam Khan, Erica Westwood, Ahmad Wesal Zaman, Joseph P. Hicks, Shahzad Ali Khan, Muhammad Amir Khan

**Affiliations:** 1 Association for Social Development (ASD), Islamabad, Pakistan; 2 University of Leeds, Leeds, UK; 3 University of Oxford, Oxford, UK; 4 Health Services Academy, Islamabad, Pakistan; 5 International Centre for Antimicrobial Resistance Solutions, Copenhagen, Denmark

**Keywords:** Primary healthcare, Antimicrobial resistance, Antibiotic prescription, Contextualised intervention, Care-cascade

## Abstract

**Introduction:**

Antimicrobial resistance (AMR) poses a major global public health threat, with deaths projected to reach 10 million annually by 2050, disproportionately affecting LMICs. Almost 80% of prescriptions at primary healthcare facilities contain at least one antibiotic. This study explores the existing care context at rural health centres (RHC) in Punjab to inform the development of an intervention package aimed at reducing inappropriate antibiotic prescriptions for two most common infections in the outpatient department of RHCs i.e., upper respiratory tract infections (URTI) and diarrhoea.

**Methods:**

This study employed a care-cascade framework, selected for its suitability in resource-constrained settings and its applicability in contexts with limited evidence on AMR and stewardship practices. The mixed-methods design included: literature review; facility review (n = 3); key informant interviews with healthcare providers (n = 6) and patients (n = 6); and focus group discussions with health managers (n = 1), healthcare providers (n = 2), patients (n = 2), patients with experience of using mobile healthcare applications (n = 2) and community significants (n = 1). The data analysed at each step informed the next step and was populated in the pre-defined care tasks considered as themes.

**Results:**

Using the care-cascade framework, four major care tasks were identified in URTI and diarrhoea management at RHCs: (1) patient consultation and laboratory testing, (2) drug prescription, (3) drug dispensing, and (4) patient education and counselling. At the RHCs there were no standard guidelines or diagnostic protocols being followed, inadequate lab facilities, and very brief patient consultations. Antibiotic prescription was largely empirical, driven by doctor’s clinical acumen and patient demands. Drug dispensing practices were inconsistent, involving dispensing partial courses without counselling. Patients were insufficiently counselled on disease management and antibiotic use. Stakeholders made context specific recommendations for developing clinical protocols, patient counselling, and training both doctors and dispensers. They also informed the design and content of the digital tools for patient engagement.

**Conclusion:**

This study underscores the complexity of antibiotic stewardship in rural health systems. A care-cascade framework allowed us to unpack these multi-layered challenges and identify leverage points for improvement. Future implementation strategies must embed these insights into program design, ensuring that stewardship is not merely a clinical responsibility but a health system imperative.

## Introduction

Every year, around 700,000 people are affected by a rising global health threat i.e., antimicrobial resistance (AMR). The estimated annual deaths caused by AMR by 2050 are likely to rise to approximately 39 million [1]. The problem of AMR is particularly worse in low-and middle-income countries (LMICs), contributing to around 85% (i.e., 4·3 out of 5 million) annual deaths [2]. In Pakistan, an estimated 59,000 deaths were directly attributable to antimicrobial resistance (AMR), while approximately 200,000 deaths were associated with it in 2019 [3].

The AMR Global Action Plan was approved at the 68th session of the World Health Assembly in 2015 and endorsed by the Government of Pakistan. As a result, Pakistan adopted the GAP-AMR as a National Action Plan (NAP) in 2016 and was enrolled in the GLASS surveillance system in 2018 [4]. The implementation of the NAP in Pakistan is supported by DAI-funded Fleming Fund Program in collaboration with the government since January 2021, and has been mainly focused on strengthening AMR and antimicrobial use surveillance systems at a macro-level, engagement of hospital-based clinicians in promoting appropriate use of antibiotics and enhancing laboratory infrastructure [5]. Despite numerous policy discussions, workshops, and official reports, implementation of effective AMR containment actions remains limited. Significant shortcomings have been identified in Pakistan’s national response to AMR five years after the launch of its National Action Plan. The situation highlights an urgent need to translate policies and dialogues into effective ground implementation [6]. Cost-effective interventions must be planned and implemented by the health authorities to lessen the burden of AMR [7].

The first core element for the development of any healthcare intervention is understanding the context [8–11]. A robust and effective intervention design involves co-development with relevant stakeholders. This collaborative process enables shared decision-making around the content, format, and delivery of the intervention, ensuring that it is tailored to the specific context i.e. social, cultural, and operational realities of the setting [12]. By grounding intervention development in contextual understanding, they are more likely to be relevant, accepted, and impactful [13, 14].

Around 80% of the antibiotics are being used in primary health care settings [15], and therefore, there is a strong recommendation by WHO to integrate AMR response into primary health care [16]. AMR response at primary health care settings in LMICs should be contextualised, engage the community, and involve the use of information and health literacy to effectively reduce AMR [17].

Since the devolution of Pakistan’s health system in 2010, healthcare has mainly been a provincial responsibility [16]. The health system of Pakistan is divided into three main structures i.e., primary, secondary and tertiary healthcare, with each province leading its health system. The primary care consists of the basic health units (BHUs), the rural health centres (RHCs), the secondary care consists of the tehsil headquarter hospitals (THQs) and district headquarter hospitals (DHQs), and the tertiary care consists of teaching hospitals [18]. The RHCs serve two-thirds of the rural population in Pakistan [19], with an average catchment population of up to 100,000 people per RHC in Punjab [20].

According to the District Health Information System (DHIS) Punjab 2023–2024 report, the most common conditions recorded in outpatient visits were upper respiratory tract infections (36.3%) and diarrhoea/ gastroenteritis (7.3%) [21]. This study aims to understand the context i.e., existing care setting and clinical practices; and inform the development of an intervention package aimed at reducing inappropriate prescription of antibiotics for upper respiratory tract infections and diarrhoea at RHCs in Punjab, Pakistan. The intervention package to be informed by the context review included: desk-aide for doctors, training manuals for doctors and allied staff, a mobile app for patient engagement and a “WhatsApp bot” for patient safety.

## Methods

The study was conducted at primary healthcare facilities i.e., rural health centres in three districts of Punjab, Pakistan. These districts were selected systematically to geographically represent the south, central, and northern regions of Punjab and randomly selected such that one district from each region i.e., Jhang (Central), Muzaffargarh (South), and Sargodha (North) is included. One rural health centre was randomly selected from each of the three districts to ensure contextual diversity while maintaining feasibility for in-depth qualitative inquiry. The study was conducted between November 2023 and May 2024.

### Study design

**Fig. 1:**
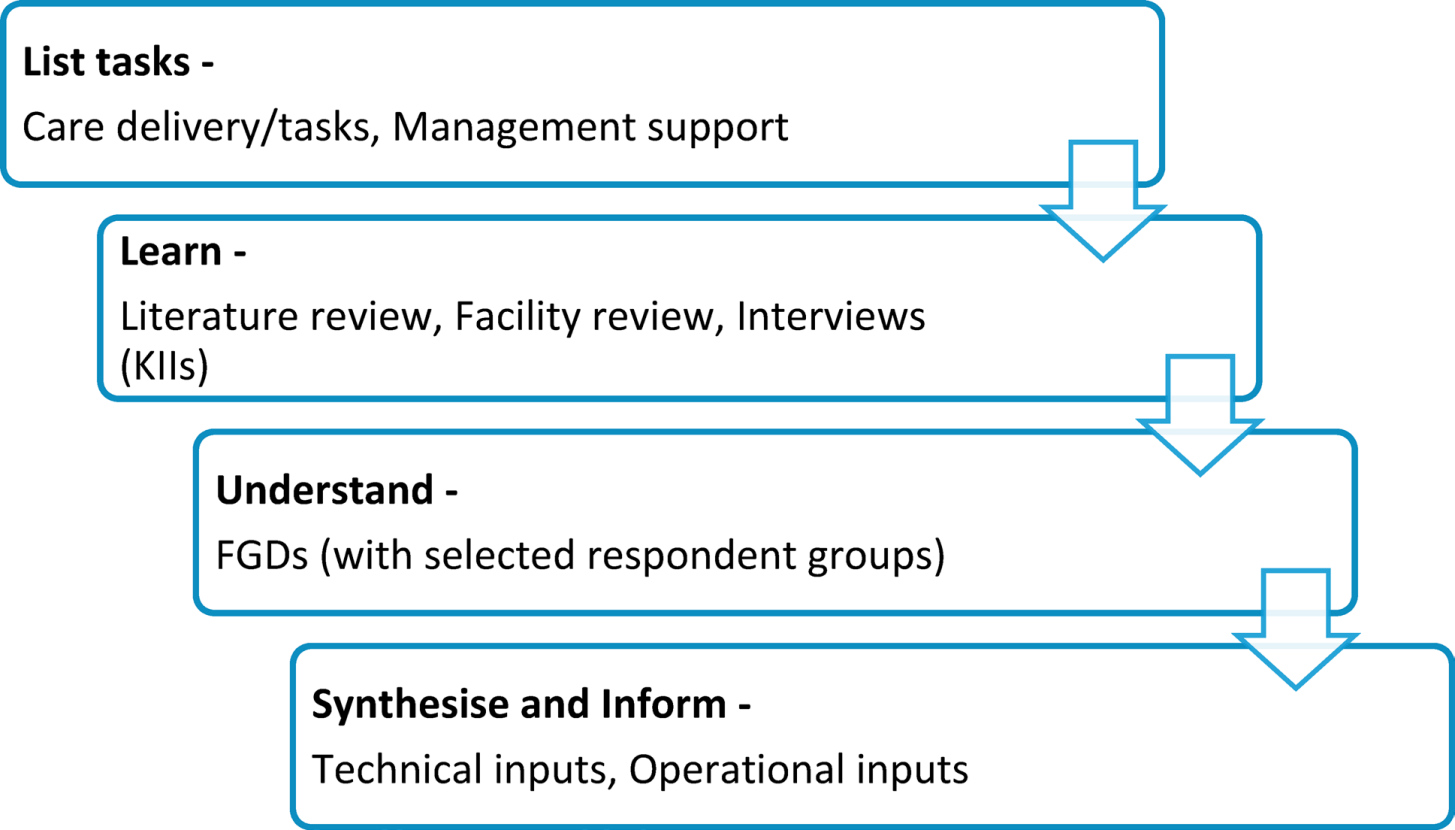
Care-task framework

This study employs the care-cascade framework (Fig. 1), a sequential multi-method approach which utilises quantitative and qualitative data collected from primary and secondary sources (to be published separately). This was selected because the utilisation of known approaches, such as the ADAPT guidance [22] and the Medical Research Council framework for complex interventions [23] in Pakistan is hindered owing to two key factors in our experience: lack of available evidence and resource constraints. Hence, we used a simplified, evidence-informed tool designed for resource-limited settings. This approach reduces reliance on specialised research expertise while remaining practical and effective.

A core expert group (CEG) comprising both technical and managerial experts and moderated by a senior public health professional was formulated to lead the process. A Technical Working Group (TWG) was established by the Directorate General of Health Services, Punjab, with representation from international organisations such as the World Health Organisation, the UK Health Security Agency, as well as the Ministry of National Health Services, Regulations and Coordination, Government of Punjab. Bimonthly meetings were held for CEG and once every two months for TWG consultations over a period of six months.

The CEG formulated actionable components of the intended intervention, which we labelled as “care tasks” (e.g., clinical diagnosis, drug dispensing, or patient counselling). In this process, intervention components [13] were conceptually broken down through implementation science perspective to list tasks for delivering and managing the care. An implementation research logic model [24] was used for this activity. The proceedings, as well as the final list of the care tasks were reviewed and endorsed by the TWG. These care tasks were subsequently used as pre-defined themes for every step of the study.

Following the care task listing, the care-cascade framework has three distinct but interrelated steps; learn, understand, and synthesise/inform the intervention, each step being informed by and expanding on the previous step. All three of these steps are employed to contextualise the key actionable components of the intervention i.e., the care tasks.

The first step is to *learn* about the existing landscape by reviewing the available evidence as well as the facility settings. The second step is to *understand* key contextual nuances through engagement with relevant stakeholders. This is followed by *synthesising* the findings and *informing* the components of the intervention. The main activities conducted to implement these steps were as follows:

- Literature review was conducted to learn from the global and national experiences of improving antibiotic prescription practices, as well as conducting antimicrobial stewardship activities at primary healthcare settings
- Facility review was done to understand the care setting and practices
- Key informant interviews were carried out to have a better understanding of the gaps identified by the healthcare staff, as well as from the literature and facility review regarding the care setting and practices at the RHC for the management of acute URTI and diarrhoea
- Focus group discussions (FGDs) were aimed at discussing and finding solutions to the problems identified from the key informant interviews and the facility review.

### Data collection

For the literature review, searches were conducted on multiple databases, including PubMed, Scopus, and CINAHL, targeting the established care tasks. In addition, Google Scholar was also used for grey literature. The duration for the literature search included all publications in the English language published from January 2005 to December 2023. A systematic set of steps was followed for this narrative literature review [25] which was then complemented by a targeted rapid literature review technique [26]. Reviewing the bibliographies of all the identified literature was helpful for further identifying relevant materials.

The facility review was done through onsite observation and review of facility records. Two mid-level researchers visited one randomly selected rural health centre in each of the three selected districts. It was conducted using a structured checklist developed to specifically address the key areas and gaps identified through the literature review relevant to the listed care tasks.

The activities and interactions of RHC staff with patients, as well as the facility records of a single working day were reviewed by both researchers and findings were noted in the checklist independently. The findings of both researchers were then reviewed by a senior public health professional and any discrepancy was discussed with both researchers, establishing a common consensus.

The facility review was complemented by key-informant interviews (KII) at the RHCs. The interview guides for the KIIs were informed by the learnings from literature while the facility review focused on the pre-defined care tasks. Separate interview guides were developed for health care providers and patients, which were then pilot tested [27] for ensuring phrasing consistency as well as construct validity and necessary changes were made for refinement. Purposive sampling method [28] was used to identify relevant stakeholders for the KIIs who included healthcare providers currently providing services (minimum 1 year experience of working in public primary health care facilities) or patients who have received healthcare services at the designated RHCs. The KIIs were conducted in Urdu with healthcare providers (n = 6) and patients with diarrhoea and/or acute respiratory infections (n = 6). The interviews were conducted by a mid-level qualitative researcher with a background in public health who was accompanied by a note taker. The duration of KIIs varied from 30 to 40 min per interview and were audio recorded and transcribed verbatim.

A total of eight FGDs were conducted with homogenous groups consisting of 4–6 participants from specific stakeholder categories. The FGDs were organised separately using tailor-made guides for each of the following groups: doctors; allied healthcare staff; district as well as provincial level health managers; male patients with diarrhoea and/or acute upper respiratory infections; female patients with diarrhoea and/or acute upper respiratory infections; male patients with experience using mobile applications for healthcare; female patients with experience using mobile applications for healthcare; and community representatives. Each FGD included only participants from the same stakeholder group to ensure open and contextually relevant discussion. The FGDs lasted between 60 and 75 min and were conducted by trained qualitative researchers in the local language to facilitate participation. Two mid-level researchers moderated the discussion and took notes.

### Data analysis

All the findings from every step were populated into the respective sections of the care task framework.

For the literature review, the evidence was consolidated into summary findings according to the guidelines provided by Snyder [29]. The observations from the health facility were compared, and summary learnings were subsequently populated in the relevant matrix cells.

For qualitative data collected in KIIs and FGDs, deductive thematic analysis [30] was conducted manually with care tasks as pre-defined themes. The transcripts from the KIIS and FGDs were independently analysed by two mid-level researchers, and codes, categories, and themes were developed from the transcripts. Afterwards, a senior researcher reviewed the analysis process and brought consensus to the process after consultation with both researchers. Lastly, the thematic findings from KIIs and FGDs were populated into the relevant column of the care-cascade framework.

Once the framework was populated, the findings from all methods were synthesised by the core expert group to formulate operational and technical recommendations for informing the healthcare delivery. These recommendations were then reviewed, endorsed, and modified according to the guidance by TWG.

### Ethical considerations

This study was approved by the Ethics Advisory Group (EAG) of the Association for Social Development (ASD) (Reference number: ASD-EAG-24-001a). Written informed consent was obtained from all participants. The confidentiality and privacy of all interviewees and participants were respected in data analysis and reporting.

### Findings

The Core Expert Group (CEG), using the care-cascade framework, outlined the essential care tasks involved in providing outpatient management of URTI and diarrhoea at the RHCs, including: (1) patient consultation and laboratory testing; (2) drug prescription; (3) drug dispensing; and (4) patient education/counselling. The findings matrix is presented in Table 1.

**Table 1:** Findings matrix

Learn				Understand	Synthesise and Inform	
Literature review	Facility review	Key informant interviews		Focus group discussions	Oper. (literature + prog.)	Tech. (global + national experts)
		Providers	Patients			
*Care Task 1—Patient consultation and laboratory testing*						
No guidelines available at the RHCs to aid the consultation and treatment Communication, improves consultation experience Lab facilities are minimal specially at primary care level Minimal OPD consultation time Modest-sized lab Commonly available tests at the RHC laboratory include blood glucose, complete blood picture (CBC) and chest X-Ray (CXR) at a subsidised rate	Daily average OPD: 150 – 250 OPD consultation time was around 2–3 min per patient No care guidelines or job-aide available History and physical exam, informed by core medical training and experience Multiple patients present at a time in the consultation room No examination couch or privacy curtains. Referral to THQ/DHQ for tests	Doctor mostly depends on presenting symptoms and patient’s explanation Tests mostly prescribed to patients in case the symptoms do not subside with the initial treatment	Preference for same gender providers Privacy is a concern in consultation Patients do prefer long consultation Compromised patient privacy during history taking and physical examination Patients prefer minimal testing prior to treatment due to their health condition and financial constraints	No plans to strengthen RHC lab for culture sensitivity testing Consultation setting change not feasible; focus on efficient consultation within the available resources Contextualised protocol/guideline, enhanced staff skills, and support for the practice	Efficient consultation using guidelines Ask RHCs to consider local feasible privacy measures Encourage allied staff communication Testing scope/ quality beyond scope; focus link with hospital labs	Job aide for history, exam and diagnosis Include signs /symptoms of bacterial infection Include detailed examination Define mild, moderate, severe URTI/diarrhoea No lab tests, unless inevitable
*Care Task 2—Drug prescription*						
WHO AWaRe guidelines MMIDSP guidelines in Pakistan Hospitals lack guidelines for empirical treatment Provider-end factors for AB prescription include: no diagnostic facility, heavy patient load, patient preferences, lack of patient follow-up, prevalent prescribing culture, and fear of M/L problems Patient-end factors for AB prescription include: denial about the self-limiting nature of their illness, as well as easy access to antibiotics from other doctors or over the counter if not prescribed Lack providers’ training on appropriate AB prescription	No guidelines available to guide prescription of ABs Limited range of ABs available in RHC stock including: Amoxicillin, Co-amoxiclav, Azithromycin, Cefixime, Ciprofloxacin, Metronidazole, and Co-trimoxazole Majority of patients get ≥ 1 drug prescribed Two-third prescriptions contain AB Drugs procured monthly by the RHC through the district health authority Allocated budget for medicines has limited flexibility for RHC staff to decide (type/ quantity) ABs selected as per need, and their quantity varies between the seasons Medicines stored in the main drug inventory Drugs provided to the OPD on a daily basis	Lack of staff training and ABs prescription audit Almost all patient with URTI/ diarrhea gets AB prescribed Prescription is influenced by stock availability Some physicians perceive over-prescription to be safer than under-prescription Patient’s preferences accounted for Patients want quick relief	Most expect a drug prescription Lack of education on AB by doctors Most trust physicians’ advice regarding ABs Most adhere to the advised treatment	Provision of empirical treatment protocols Training to enhance the staff skills Monitoring of AB prescriptions and stock Advised prescription should be explained to patients	Tailor-made desk-aide to promote appropriate prescription Train both doctors and allied HC staff Inform empirical treatment with ABs procured	Train in conservative care Define empirical AB therapy (1st/2nd line) in the guidelines Include guidelines on follow-up care as required
*Care Task 3—Drug Dispensing*						
ABs dispensed at private pharmacies without prescriptions Customers highly demand availability of ABs over the counter	ABs are dispensed for 2 days, patient revisits to receive complete course RHC dispense around ≥ 500 AB courses per month Dispensers counsel patients on intake of prescribed drugs	Two-day drug dispensing practice due to limited drug stock, patient adherence, and side-effects Doctor updated about the drug stocks; and prescribes accordingly	Patients mostly receive prescribed drugs free-of-cost Quality of drugs is acceptable to most patients Women and elderly patients find revisiting OPD challenging to collect complete course of antibiotics	Improve the drug dispensing records (train the staff & monitor) Explore ways to retrieve patients for complete AB course Make easier for patients to get ABs dispensed on revisit Provider encouraged & monitored to not prescribe unavailable ABs	Dispensing full 5-day course not possible, encourage 3-day dispensing; also strengthen patient education for adherence Consider making subsequent dispensing easier on revisit	Doctors to be trained in advising 3-day course of ABs Dispense drugs/ dosage according to prescription
*Care Task 4—Patient education*						
Patient education regarding disease prevention and reducing the misuse of antibiotics is important in URTI and diarrhoea Educating patients through digital smartphone applications is considered an effective mechanism for improving their knowledge, treatment adherence, satisfaction, and clinical outcomes	No standardised protocols/messages available for education or reporting complaints Short consultation timings affect patient counselling Lack of patient counselling guidelines Time constraints for doctors to counsel patients Dispenser educates patients for other diseases	Many providers perceive that patients are generally not keen to get educated regarding the disease Time constraint for doctors Efficient use of time may include appropriate messages and staff skills	Patients do feel that currently they receive limited information about the disease and its management Patients find it difficult to remember the education messages Patients perceive need for an accessible knowledge	Contextualized messages to make the counseling efficient Take-home materials with key messages Online access to knowledge Pictorial messages, take-home material, online access to knowledge	Develop education material Include responsible consumption Enable allied staff for patient education Supplement in-person counselling; posters, digital app and take-home material	Adapt URTI educational messages from CDC, NHS Adapt diarrhea educational messages from WHO, NHS and World Gastroenterology Organization Global Guidelines

### Patient consultation and laboratory testing

Review of the literature and facilities showed that there were no guidelines available at the RHCs to aid the consultation and treatment recommendations for patients presenting with URTI or diarrhoea [31]. History taking and physical examination of the patients was only informed by the doctors’ core medical training and hands-on experience of care delivery. The average OPD consultation time was around 2–3 min per patient due to heavy patient load. The consultation rooms mostly had multiple patients present at a time and lacked an examination couch or privacy curtains. Interviews revealed that this resulted in compromised patient privacy during history taking and physical examination.

I found it very hard to explain my symptoms to the doctor openly because many other people who were present during the consultation were listening. The doctor was a male, which also made me shy. (Female with diarrhoea, KII)

According to the literature, there are minimal lab services in terms of diagnostic breadth and non-existent drug sensitivity testing at the primary care level in Pakistan [32, 33]. The RHCs had a modest-sized laboratory room (accommodating 4–5 people at a time). Commonly available tests at the RHC laboratory included blood glucose, complete blood picture (CBC) and chest X-Ray (CXR) at a subsidised rate. For tests not available at the RHC, patients were referred to the tehsil headquarter (THQ) or the district headquarter (DHQ) hospitals. Interviews showed that tests are mostly prescribed to patients in case the symptoms do not subside with the initial treatment. Patients also preferred minimal testing prior to treatment due to their health condition and financial constraints.

We do not have any clear guidelines for proper history taking and clinical examination to diagnose correctly without test results, and to decide whether or not to prescribe antibiotics (Male doctor, KII)

The FGDs highlighted a clear need for contextualised protocols/guidelines for efficient diagnosis and management of URTI and diarrhoea.

The doctors are experienced; however, a large influx of patients and minimal consultation time is a risk for inconsistent care. Therefore, there is a need for standardised consultation guidelines to be in place. We need clear, practical tools to streamline clinical decision-making. (Provincial program manager, FGD)

Some of the focus areas that were suggested to be addressed in the guidelines/protocols were for improving clinical diagnosis of URTI and diarrhoea, to clinically differentiate between viral and bacterial infections, to assess the severity of conditions, and to sensitise the healthcare staff on patients’ privacy during consultation. The managers and doctors further emphasised the need for training of the healthcare staff on these developed protocols/guidelines.

Significant strengthening of RHC lab, including C/S services, is not a part of any current plan. Therefore, protocols, and doctors’ clinical skills and practices may be enhanced in terms of prescribing for empirical treatment. (District level manager, FGD)

### Drug prescription

Review of literature revealed that the World Health Organisation (WHO) has published guidelines (AWaRe) for the appropriate prescription of antibiotics [34]. Furthermore, clinical guidelines have been developed by the Medical Microbiology & Infectious Diseases Society of Pakistan (MMIDSP) for the management of common infectious diseases [35]. However, MMIDSP guidelines do not provide guidance to distinguish viral from bacterial URTI and diarrhoeal infection. Therefore, there is a need for guidelines to guide empiric therapy in Pakistan [36]. The review of facilities also found no specific protocols/guidelines regarding drug prescription for URTI and diarrhoea available at RHCs. A limited range of antibiotics was available for procurement by the RHCs to cover commonly occurring infections (see Table 2 for commonly available antibiotics at RHCs). Drugs were procured monthly by the RHC through the district health authority. A budget was allocated to the RHCs for the procurement of antibiotics with limited flexibility of reallocation. The antibiotics were selected as per need, and their quantity vary between the seasons (e.g., antibiotics for respiratory infections are stocked in larger quantities during the winter season). Medicines were stored in the main drug inventory at the RHC and provided to the OPD dispensary on a daily basis as per the need. Facility review also showed that most patients got prescribed ≥ 1 drug, and about two-thirds of all prescriptions contained antibiotics.

**Table 2:** Common available antibiotics at RHCs

Generic name	Dosage
*Amoxicillin*	500 mg
*Co-amoxiclav*	625 mg, 156.25 mg/ml
*Azithromycin*	500 mg
*Cefixime*	500 mg, 100 mg/5 ml, 200 mg/5 ml
*Ciprofloxacin*	500 mg, 125 mg
*Metronidazole*	500 mg, 400 mg, 200 mg/5 ml, 120 mg/ml
*Co-trimoxazole*	160/800 mg, 80/400 mg/5 ml

Review of the literature revealed that the prescription of antibiotics is influenced by multiple provider and patient-related factors. The provider-related factors leading to antibiotic prescription included lack of diagnostic facility, workload, perceived patient preferences, lack of patient follow-up [37], prevalent prescribing culture [38], and fear of medicolegal problems if the patient’s health deteriorated [39]. The patient-related factors included denial about the self-limiting nature of their illness, as well as easy access to antibiotics from other doctors or over the counter if not prescribed [40, 41]. These considerations were further validated through the facility review. In the interviews, the doctors shared that their prescription was based on their clinical acumen; however, some additional influencing factors were drug availability and the patient’s preferences for certain drugs they perceive to give quick relief.

Patients often don’t know whether a drug is an antibiotic or not. However, they do recognise the names or appearances of the drugs that they feel have worked for them previously. So, they say, for example, ‘give me Amoxil’ or ‘prescribe me that green coloured capsule.’ (Male doctor, KII)

The managers and providers underscored a need for protocols/guidelines for appropriate antibiotic prescription for URTI and diarrhoea (based on clinical examination) and to treat the disease within given resources (i.e., available drugs). They also suggested that the guidelines/protocol should include instructions regarding conservative care in case of viral infections, as well as follow-up care for the patients.

### Drug dispensing

Review of facilities found that a qualified dispenser (a 2-year formal training on drug dispensing) is responsible for dispensing the drugs, counselling the patient on intake of prescribed drugs and maintaining the records. The records showed that the drugs (particularly antibiotics) are usually dispensed for up to two days, and patients are asked to visit the RHC again for the remaining dose. When further explored through interviews, it was found that dispensing drugs for two days relates to limited stock of drugs, risk of side-effects and uncertain patient adherence to drug intake. The interviews also showed that the patients were inadequately counselled regarding the importance of collecting the remaining dose, and therefore, did not always adhere to this protocol.

In the FGDs conducted, dispensing a complete course of antibiotics was encouraged. However, in given circumstances, it was suggested that adherence to treatment despite partial dispensing should be enhanced through standardised patient education on responsible consumption (i.e., dosage, treatment duration, follow-up and not getting antibiotics prescribed from elsewhere). Since in RHC settings, dispensers educate the patients regarding the prescribed treatment, it was suggested that they should be enabled to provide the standardised education to the patients. The importance of continued support mechanisms to ensure the provision of quality care was also emphasised.

We should explore the possibility of dispensing a full course of antibiotics to everyone. Currently, we dispense the full course of antibiotics (i.e., for 7 days) to only those who we deem absolutely necessary. (Female doctor, FGD)

### Patient education

A review of the literature showed the significance of patient education in the case of respiratory infections [42] and diarrhoea [43] regarding disease prevention and reducing the misuse of antibiotics. Educating patients through digital smartphone applications is considered an effective mechanism for improving their knowledge, medication or treatment adherence, satisfaction, and clinical outcomes [44]. The facilities’ review showed that there were no standardised protocols available for education or reporting complaints of URTI and diarrhoea patients. Due to minimal consultation time, the doctors provided inadequate counselling regarding URTI/diarrhoea disease, it’s treatment and prevention. It was also observed that the patient education for other diseases, e.g., tuberculosis, diabetes etc. was carried out by the RHC dispensers.

If patients have an emergency and want to reach out, there are only two ways; either they visit the RHC directly or call Rescue 1122 (ambulance service) (Female doctor, KII)

The dispensers felt a need for having a standardised mechanism in place for on-site education and further suggested that patients should be efficiently educated through easy-to-understand and concise messages. They further stressed the importance of a source to make information accessible at home. Since the doctors have limited consultation time per patient, one of the strategies suggested to address this issue was to train the dispenser to educate the patients. The managers expressed a need for a mechanism to not only educate but also engage patients in making care more responsive to their needs.

Since the time is limited, it would be better if the information were available at home, where people could read it themselves. It should be easy to understand because there would be no healthcare staff at home to guide. (Female with URTI, FGD)

The use of healthcare mobile applications for engaging patients was found feasible and easily accessible. The existing users of these apps found some features to be significantly useful including audio assistance throughout the app and pictorial depiction of the messages for less literate people. Some users expressed that being able to access information with compromised internet access will further improve useability. A structured platform for participation was advocated by the community representatives who had previously been a part of such platforms where they were able to generate possible solutions, in collaboration with the doctors, in response to challenges reported by patients regarding care delivery for other conditions e.g., hypertension.

We’ve had a proper platform to sit together before, like with the hypertension project, people actually shared what was going wrong, and we came up with practical solutions along with the doctors. That kind of space really helps. We need something similar now too. (Male with experience of digital health application, FGD)

### Synthesise and inform the intervention package

Based on the literature and data collection processes within this context review, there is a need for guidelines to inform the diagnosis and treatment of URTI and diarrhoea, given the limited facilities and skills available at the RHC level. Some of the key areas that should be covered in the guidelines are clinical diagnosis of bacterial and viral infections (given the absence of lab diagnostics) as well as its severity; the required treatment; and follow up. The tailor-made desk-aide for the doctors to help them diagnose and treat efficiently will cover these details.

Educating patients and engaging them to make care responsive to their needs is also an essential component in AMR response. The dispenser who is already counselling patients for other diseases such as TB and NCDs, should also educate URTI and diarrhoea patients on care i.e., prevention and treatment. To enable the dispenser to provide standardised education, the patient counselling flipbook will include easily understandable pictorial-aided messages focusing on: understanding URTI/diarrhoea disease; conservative management of the disease; prevention; and risk of AMR (including what causes it and how to avoid it). In addition to educating patients at facilities, a need for a source of knowledge that is accessible even after the facility visit was also validated. This will be addressed through a digital mobile application that will not only make knowledge accessible to patients at all times but will also provide them with awareness about their rights as patients.

Furthermore, the application will also be able to support the engagement of patients in making care responsive to their needs. The application will provide the patients with a platform to report care and social challenges. The care challenges will be addressed through patient-provider forums that will be conducted at each facility (based on care challenges reported at the respective facilities), and the social challenges will be addressed through peer support videos that will be incorporated within the application.

Successful implementation of these intervention components will require adequate training of healthcare staff i.e., doctors and allied staff (i.e., dispensers). Therefore, there is a need for training manuals that covers all intervention components in detail with clear instructions on roles. The doctors will be trained on efficient diagnosis; treatment; and prescription writing (i.e., diagnosis, drug, dose and duration). The allied staff will be trained on counselling the patients and guiding patients about installation as well as the use of the mobile app and WhatsApp bot.

## Discussion

This study reviews the context to inform an intervention package for URTI and diarrhoea care at primary healthcare facilities in Pakistan. The study has used the care-cascade framework to map key systemic, structural, and behavioural factors influencing antimicrobial use. By organising care delivery into care tasks, which are: patient consultation and laboratory investigations; drug prescription; drug dispensing; and patient education, we were able to identify points of vulnerability that contribute to inappropriate antibiotic use, and opportunities for contextually relevant stewardship interventions.

Our study confirms that the patient consultations are brief (typically lasting only 2 to 3 min) due to heavy patient resulting in compromised care and patient counselling. The laboratory investigations are compromised due to a range of contextual challenges, resulting in clinical decision-making that relies more on provider intuition. Evidence from a systematic review of 67 countries highlights significant global variation in the average length of primary care consultations. Notably, in 18 countries representing approximately half of the world’s population, the mean consultation duration is reported to be five minutes or less. Such limited time for patient interaction raises serious concerns regarding the quality of care. The systematic review also confirms that in some countries, including Pakistan, individual primary care providers may conduct over 90 consultations per day [45].

This study found that in the face of diagnostic uncertainty, with lack of laboratory tests, providers often defaulted to empirical broad-spectrum antibiotic prescriptions as a precautionary measure. This practice is widely reported across LMICs including India and within Africa where diagnostic and lab testing capacity at public primary healthcare set-ups are a constraint [46, 47]. The study also found that there is lack of standard guidelines at the primary healthcare centres, which is consistent with other studies in southern Punjab [48] and Northern Balochistan, Pakistan which saw that specific instruction on the management of URTIs is not available in Pakistan [35]. The absence of accurate diagnostics without the confirmatory laboratory test and without any standard guidelines, represents a significant missed opportunity for improvements in targeted therapy and antibiotic stewardship [49].

Our study also found that counselling for patients was completely absent, and antibiotics were prescribed but full course were in most cases not dispensed for patients, instead opting to dispense only partial treatments. This contributes to misuse or over-use of antibiotics from over-the-counter distribution from other local pharmacies, which is directly leading to resistant microorganisms [50]. Similarly, a narrative review has shown that pharmacist-led interventions, including educational counselling regarding the prescription can reduce inappropriate antibiotic use significantly [51]. A systematic review exhibits that educational counselling, when integrated with supporting patient educational material enhances the quality of the counselling [52]. Strengthening the patient education and engagement can reinforce appropriate medicine use in primary healthcare system.

In summary, inappropriate antibiotic use in rural LMIC settings stems from complex, interrelated factors across multiple stages of care. Our study underscores the need for integrated contextualised interventions, that address care tasks in outpatient settings ensuring comprehensive assessment at consultation, accurate diagnostics and appropriate case management according to standardised guidelines.

The strength of this study is the use of care-cascade framework offers a comprehensive approach to systematically understand the context using multiple methods and intervene in the care tasks, ultimately contributing to the containment of antimicrobial resistance and improvement of primary healthcare quality. This framework can be used to further inform the wide-scale application of the care-task framework across different and unique contexts, while applying a simplified and systematic method. The limitations of the study are that it focuses on understanding the context specific to only two infectious conditions i.e., URTI and diarrhoea and has a small sample size i.e., very few numbers of facilities. Therefore, the findings may not be necessarily generalisable.

### Recommendations

To achieve meaningful and sustained improvements in care quality and antibiotic stewardship, policymakers should prioritise addressing some of the systemic aspects of care provision at RHCs such as managing staff workload, ensuring gender-sensitive workforce deployment, and addressing dispensing mechanisms at the primary healthcare level.

## Conclusion

This study underscores the complexity of antibiotic stewardship in rural health systems, where each step of the care task is affected by structural, behavioural, and systemic constraints. Strengthening diagnostic integration, expanding role of allied staff, improving patient-provider communication, and instituting patient education are all essential for appropriate antibiotic use.

The care-cascade framework allowed us to unpack these multi-layered challenges and identify leverage points for improvement. Future implementation strategies must embed these insights into program design, ensuring that stewardship is not merely a clinical responsibility but a health system imperative.

## Data Availability

The data generated and analysed during this study are available with the authors and can be provided upon reasonable request.
